# Functional characterization of three G protein-coupled acetylcholine receptors in parasitic nematode *Trichinella spiralis*

**DOI:** 10.1016/j.ijpddr.2023.11.005

**Published:** 2023-11-24

**Authors:** Cáinà Nìng, Aurélie Heckmann, Lourdes Mateos-Hernández, Grégory Karadjian, Ladislav Šimo

**Affiliations:** Laboratoire de Santé Animale, UMR BIPAR, Ecole Nationale Vétérinaire d’Alfort, INRAE, ANSES, F-94700 Maisons-Alfort, France

**Keywords:** Trichinella, GAR, CHO cell, Calcium mobilization, Pharmacology

## Abstract

The physiological significance of metabotropic acetylcholine receptors in parasitic nematodes remains largely unexplored. Here, three different *Trichinella spiralis* G protein-coupled acetylcholine receptors (TsGAR-1, -2, and -3) were identified in the genome of *T*. *spiralis*. The phylogenetic analyses showed that TsGAR-1 and -2 receptors belong to a distinct clade specific to invertebrates, while TsGAR-3 is closest to the cluster of mammalian-type muscarinic acetylcholine receptors (mAChR). The mRNA of TsGAR-1, -2, and -3 was detected in muscle larvae, newborn larvae, and adults. The functional aequorin-based assay in Chinese hamster ovary cells revealed that all three types of *T. spiralis* GARs trigger the G_q/11_ pathway upon activation of the receptor with the acetylcholine ligand. TsGAR-1 and TsGAR-2 showed atypical affinity with classical muscarinic agonists, while TsGAR-3 was sensitive to all muscarinic agonists tested. High concentrations of propiverine antagonist blocked the activities of all three TsGARs, while atropine and scopolamine antagonists effectively inhibited only TsGAR-3. Our data indicate that the distinct pharmacological profile of TsGAR-1 and -2 receptors, as well as the phylogenetic distance between them and their mammalian orthologs, place them as attractive targets for the development of selective anthelmintic drugs interfering with nematodes’ cholinergic system.

## Highlights

• *Trichinella spiralis* possesses three functional G protein-coupled acetylcholine receptors (GAR-1, -2, and -3).
• All *T. spiralis* GARs couple preferentially with G_q/11_ when expressed in Chinese hamster ovary cells.
• GAR-1 and GAR-2 showed atypical affinity to muscarinic ligands.
• GAR-3's pharmacology is similar to that of mammalian muscarinic-acetylcholine receptors.

## Graphical abstract

**Figure:**
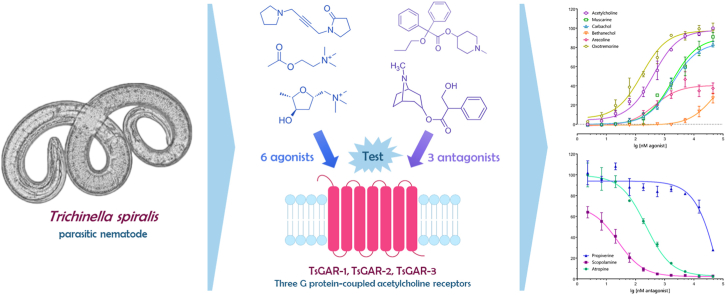

## 1 Introduction

There is a global need to effectively manage parasitic nematodes as they infect a wide range of species, and not only affect human health but also severely impact livestock and crop production (Hotez et al., 2014). *Trichinella spiralis* is a foodborne parasite that can infect humans via consumption of raw or undercooked meat, mainly pork, horse or game meat, and cause a zoonotic disease known as trichinellosis (Djurković-Djaković et al., 2013; Taha et al., 2022). Although nowadays a massive global drug application program has been well established to control parasitic nematodes, several critical points such as emerging resistance, potential health risk to vulnerable groups, and sustainable long-term profit, remain to be defined (Humphries et al., 2012). In addition to these factors, current treatments for trichinellosis are inadequate mainly due to ineffectiveness in eliminating the encysted larvae of *Trichinella* (Pozio et al., 2001).

Cell signaling, which is maintained by neurotransmitters and the cascade triggered by G protein-coupled receptors (GPCR), is an unexploited source of novel antihelminth drug targets (Kruse et al., 2014). Cell-to-cell communications via the neurotransmitter acetylcholine (ACh) regulate a variety of physiological functions in the neuronal and non-neuronal tissues of both simple or higher animals (Hannan and Hall, 1993; Wessler and Kirkpatrick, 2008; Picciotto et al., 2012). ACh signal transduction is maintained either via the ionotropic nicotinic ACh receptor (nAChRs) or the metabotropic muscarinic ACh receptor (mAChRs) (Miyazawa et al., 2003; Kruse et al., 2014). mAChRs in the GPCRs family have been intensively studied in mammals, but this field of research is progressing only slowly in invertebrates. In mammals, five different mAChRs (M1-M5) have been identified, of which M1, M3, and M5 are known to be associated with the G_q/11_ pathway leading to intracellular calcium increase, while M2 and M4 are known to preferentially couple with the G_i/0_ pathway inhibiting the intracellular cAMP level (McKinney et al., 1991; Wess, 1996; Nathanson, 2000).

It is not surprising that, likely due to the druggable feature of mAChRs in therapeutic research (Kruse et al., 2014), the nematode community was the first among invertebrate research groups to advance research in this area, thus providing a stepping stone for detailed exploration of the mAChR in other invertebrates. In *Caenorhabditis elegans*, three G protein-linked ACh receptors (GAR-1, -2, and -3) have been functionally identified (Hwang et al., 1999; Lee et al., 1999, Lee et al., 2000). These studies suggest that GAR-1 and GAR-2 are sequence-wise similar to M2 and M4 and preferentially couple with the G_i/0_ family (Lee et al., 1999, Lee et al., 2000), while GAR-3 is closer to M1, M3, and M5, and preferentially activates the G_q/11_ pathway (Hwang et al., 1999). Interestingly, GAR-1 and GAR-2 distinctly differ in their binding affinity with classical nonselective muscarinic drugs, with some differences that also exist among these two receptor types, whereas GAR-3 has a conventional pharmacological profile similar to mammalian muscarinic receptors (Hwang et al., 1999; Lee et al., 1999, Lee et al., 2000; Kimber et al., 2009). Furthermore, two types (type A and B) of mAChRs have been functionally identified in arthropods; type A (mAChR-A) has been found to have the same pharmacological profile as a mammalian group of receptors, while type B (mAChR-B) appears to have a selective affinity with classical muscarinic drugs (Collin et al., 2013). Therefore, based on this classification, both nematode GAR-1 and GAR-2 receptors were assigned to type B mAChRs, while GAR-3 belongs to type A. In a subsequent study, the same research group suggested that, multiple pharmacologically-conserved type A mAChRs exist in deuterostome phyla, while the protostome conserves only one type A. However, it has also been suggested that there are a range of type B muscarinic receptors (Ren et al., 2015) across different protostome phyla. In addition, based on shared features in certain sequence segments among mammalian M2, M4, and type B mAChRs, the authors suggested that all protostome type B receptors may couple with the G_i/0_ pathway (Ren et al., 2015).

The current study investigated the functional properties and pharmacology of three different *T. spiralis* GAR receptors transiently expressed, along with the aequorin reporter, in CHO cells. The temporal expression profile of the receptor's transcripts was also investigated in different developmental stages of *T. spiralis*. Our data shed light on nematode GARs and suggest their further exploration for developing new-generation anti-helminth drugs.

## 2 Materials and methods

### 2.1 Ethical statements

All animal experiments were carried out in accordance with EU Directive 2010/63/EU and French legislation, namely Decree no. 2013–118 of February 1st, 2013 issued by the French Ministry of Agriculture, Agri-food and Forestry. The ethical committee (C2EA-16 Comité d’éthique ComEth ANSES/ENVA/UPEC) approved all experiments under the following approval number: saisine 12–0048, ComEth 13/11/12–4.

### 2.2 Sequence analyses and phylogeny

BLAST searches of invertebrate genomes or transcriptomes were performed using NCBI databases (www.ncbi.nlm.nih.gov). Full-length open reading frames (ORFs) of putative TsGAR-1, -2, and -3 were identified by comparing via BLAST *C. elegans* orthologous GAR protein sequences (Hwang et al., 1999; Lee et al., 1999, Lee et al., 2000) against the *T. spiralis* genome sequence (Mitreva et al., 2011). The hits with highest homology were further investigated manually to identify the translation initiation signal and stop codon. For phylogenetic analysis, the ClustalW program from MEGA11 software was used to align the full protein sequences, followed by application of the neighbor-joining tree method, with 500 bootstrap replications (Tamura et al., 2021). Alignment and identity/similarity comparison of nematode GAR-1, -2, and -3 protein sequences were carried out using the MegAlignPro 17.3 tool in DNASTAR (www.dnastar.com). The transmembrane segments in GAR-1, -2, and -3 were predicted using TOPCONS software (https://topcons.cbr.su.se/pred/).

### 2.3 Collection of *T. spiralis*

*T. spiralis* ISS004 nematodes were maintained by several passages in 5-week-old female OF-1 mice (Charles River laboratories, France). The mice were maintained under specific pathogen-free conditions in individual closed cages on a ventilated rack. Infected mice carcasses were artificially digested to obtain *T*. *spiralis* muscle larvae as previously described (Gamble et al., 2000). A new batch of mice was given 1500 muscle larvae orally. After five days, the mice were euthanized and the small intestines were isolated at 37 °C in petri dishes containing pre-warmed phosphate-buffered saline (PBS) with an additional 200 UI/mL of penicillin/streptomycin (P/S) (Dominique Dutscher, France). The intestines were then cut longitudinally into 2-cm-long pieces and washed in the PBS before being transferred to a second Petri dish also containing pre-warmed PBS with P/S. The contents of the first and second petri dishes were deposited on a sieve of 200 μm and 315 μm respectively in crystallizers containing PBS with P/S and incubated for 90 min at 37 °C in 5% CO_2_. The filtrates were then respectively passed through one or two surgical gauze compresses placed on the top of funnels. Adults were retrieved 30 min later from the base of the funnels (Zocevic et al., 2011). To collect newborn larvae, harvested adult worms were incubated in pre-warmed serum-free RPMI 1640-Hepes 25 mM medium (Gibco, France) in 25 mL flasks (Dominique Dutscher, France) complemented with 2 mM L-glutamine (Dominique Dutscher, France), sodium pyruvate 1 mM (PanBiotech, France), 100 U/mL penicillin and 100 μg/mL streptomycin at 37 °C under 5% atmospheric CO_2_ for 48 h. After incubation, newborn larvae delivered by adult female worms were isolated from the culture medium by a 40 μm cell sieve. They were formed into pellets by centrifugation at 13,000 g, 4 °C for 10 min. All stages were frozen at −80° prior to RNA extraction.

### 2.4 Stage specific real-time reverse transcriptase PCR (qRT- PCR)

Total RNA from *T. spiralis* newborn larvae, muscle larvae and adults was extracted with the RNAqueous – Micro Kit® (Ambion, France). A first step of worm disruption was performed. Isolated adult worms or developmental stages muscle larvae or newborn larvae were placed in VK05 tubes (Ozyme, France) with lysis buffer and crushed in a bead beater (Precellys, France) using three crushing cycles at 6500 rpm for 15 s with breaks of 10 s. The tubes were then rinsed with an additional 50 μL of lysis buffer. The manufacturer's protocol was then followed. To avoid contamination of extracted RNA, the samples were treated with amplification-grade DNase I (Thermo Fisher, France) and frozen at −20 °C until used for reverse transcriptase PCR. The obtained RNA was reverse transcribed to cDNA using a Maxima Reverse Transcription Kit (Thermo, France) and used for RT-PCR.

Gene-specific primers for TsGAR-1, -2, and -3, and Eukaryotic translation initiation factor 3 subunit C (eif3C) (Zhang et al., 2012) were designed using AmplifX 1.7.0 b y Nicolas Jullien; CNRS, Aix-Marseille Universite - http://crn2m.univ-mrs.fr/pub/amplifx-dist and Oligo Calc (Kibbe, 2007) as follows: *gar-1*, 5′-AGTGATGTGTACGATCCGTT-3’/5′-CATGAGGTGCTTGTATGTG-3’; *gar-2*, 5′-TCATCACAAATTACACCTGAGC-3’/5′-CAGCTGAGGATGATTGCTTAG-3’; *gar-3*, 5′-TGTCACCTTCAACTGCAGT-3’/5′-GTCACAGTAATGCGCGTTTT-3’; eif3C, 5′-AACGATTAGCATCAATGTTTGACCT-3’/5′-GGTCAACTGCAAAGCTAACGTG-3’. RT-PCR was performed using the Luminaris Color HiGreen low ROX qPCR Master Mix 2*X* kit (Thermo, France) in a LightCycler 480 (Roche Diagnostics, France). The cycle consisted of an initial incubation of 10 min at 95 °C, then 40 amplification cycles of 10 s at 95 °C, 10 s at 60 °C, then 30 s at 72 °C, during which the fluorescence data were collected. qRT-PCR was performed using a Luminaris Color HiGreen low ROX in LightCycler 480 II (Roche) in two technical and two biological replicates. Relative expressions were determined to eif3C using the 2^-ΔΔCT^ method (Livak and Schmittgen, 2001) with the muscle larvae group as reference. ΔΔCt values were calculated in Microsoft Excel.

### 2.5 Gene synthesis and cloning

Putative ORF sequences of TsGAR-1 and TsGAR-2 were chemically synthetized (Biomatik Cambridge, Canada). A codon optimized sequence for heterologous amino acid sequence expression in human embryonic kidney cells was inserted into a pcDNA3.1 (+) expression vector (Invitrogen), following the addition of a Kozak site before the translation initiation codon (Biomatik Cambridge, Canada). The predicted full-length ORF of TsGAR-3 (XP_003380401.1) was amplified from cDNA of muscle larvae from *T. spiralis* strain ISS004 (see section 2.4 in Material and methods) using the primers 5′-TTCAGGAGAGATGTTGCGTT-3’ (forward) and 5′-CAGCTTGAACATTAACGACT-3’ (reverse). The PCR product was cloned using pGEM®-T Easy Vector (Promega, Madison, WI) and sequenced (Eurofins), then transferred to expression plasmid pcDNA3.1 (+) (Invitrogen) using Ecor I (New England Biolabs) restriction sides. To obtain a large amount of *T. spiralis* GAR-1, -2, -3/pcDNA3.1 (+) constructs for functional assays on the receptors, a Midiprep purification system (Qiagen) was used to isolate plasmid DNA from *E. coli* cultures.

### 2.6 Functional assays on the receptors

*T. spiralis* GARs were heterologously expressed in CHO–K1 cells (Sigma) along with the aequorin reporter—with or without the wild-type human G protein alpha 15 subunit (G_α15(16)_) (cDNA Resource Center, Bloomsburg University of Pennsylvania)—to observe activation of the receptor by monitoring bioluminescence triggered by the mobilization of intracellular calcium (Offermanns and Simon, 1995; Park et al., 2002). Therefore, there were two types of transient expression: i) TsGAR-1, -2, or -3/aequorin/G_α15(16)_; and ii) TsGAR-1, -2, or -3/aequorin. The use of G_α15(16)_ in the first assay is due to its highly efficient way of linking calcium mobilization signaling pathways to GPCRs that are preferentially coupling with G_i/o_ or G_s_ protein members (Offermanns and Simon, 1995).

For handling cell lines, all transfection details, and monitoring receptor activities, we followed the previously published study (Šimo et al., 2011, 2013; Mateos-Hernández et al., 2020). Briefly, CHO–K1 cells were kept in growth medium (Ham's F12 Media with 10% fetal bovine serum) until 70% of confluence was reached. FuGENE® HD Transfection Reagent (Promega) was then used to transfect the plasmid constructs. Prior to the assay, the cells were pre-equilibrated with coelenterazine h (Promega) for 3 h at room temperature. The assay was performed in opaque 96-well microplates (Nunc) using either Fluostar Omega (BMG Labtech) or GloMax® Plate Reader (Promega). In agonist assays, 50 μL of various doses of agonistic ligands were added to wells in triplicate, followed by injection of 50μL/∼15,000 of CHO–K1 cells. Immediately after the cell injections, luminescence values were monitored for 25 s and their integrated values over time were normalized to the largest positive control response in each plate (45 μM ACh) after background noise was subtracted. When performing the antagonist assay, cells were pre-incubated with different doses of antagonistic ligands for 5 min in RT (in a 96-well plate) and subsequently a 50 μL of 10 μM ACh solution was injected into the wells. The changes in luminescence were measured for 25 s immediately after injecting ACh, and time integrated values were normalized to the response of those wells in each plate that only contained cells with medium. A dose-response curve was plotted afterwards for each data set and half-maximum response values (EC_50_ or IC_50_) along the Hill coefficient (n_H_) were calculated using GraphPad Prism 8.0 (GraphPad Software). Two and three biological replicates were performed for the testing of *T. spiralis* GARs with or without the G_α15(16)_ and agonistic or antagonistic assay, respectively, with three technical replicates for each ligand. In terms of negative controls, a mock transfection was performed using empty pcDNA3.1 (+) plasmid, with an aequorin reporter and G_α15(16)_ subunit. To confirm the specificity of *T. spiralis* GAR-1, -2, and -3 affinity, we tested dopamine, octopamine and norepinephrine chemical agents.

The chemicals used in this study were acetylcholine chloride (Sigma A6625), (+)-muscarine chloride (Sigma M6532), carbamoylcholine chloride (Sigma C4382), bethanechol chloride (C5259), arecoline hydrobromide (Sigma 31,593), oxotremorine M (Sigma O100), atropine (Sigma A0132), propiverine hydrochloride (Sigma SML0602), (−)-scopolamine hydrobromide trihydrate (Sigma S1875), pilocarpine hydrochloride (Sigma P6503), dopamine hydrochloride (Sigma H8502), (±)-octopamine hydrochloride (Sigma O0250), and (±)-epinephrine hydrochloride (Sigma E4642).

## 3 Results and discussion

### 3.1 Three different GARs in the *T. spiralis* genome sequence

**Fig. 1:**
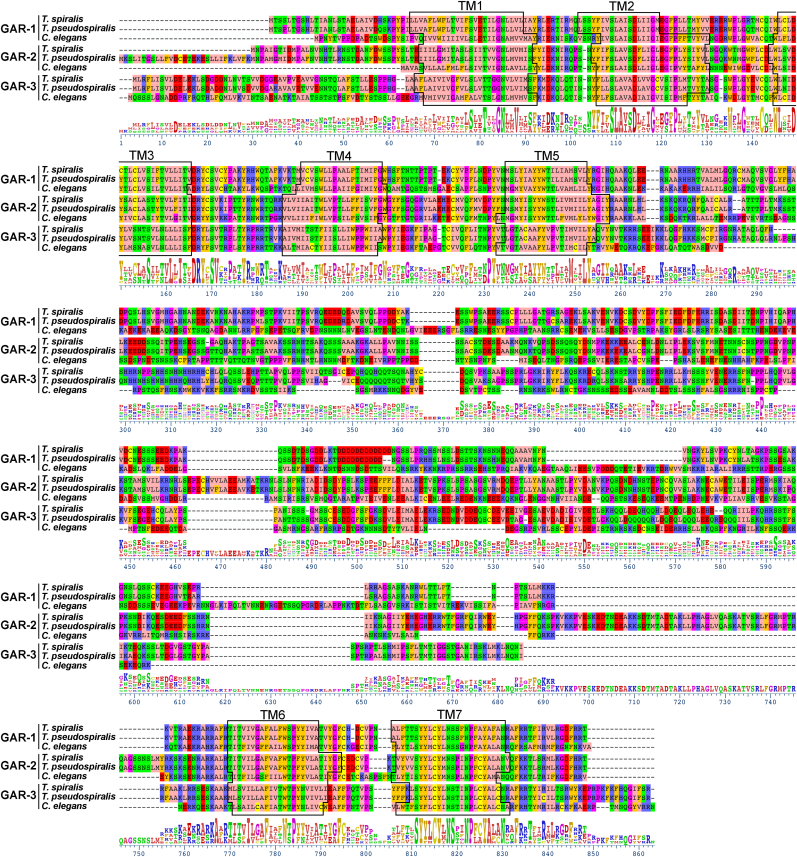
Alignment of predicted translations of the three nematode GAR types (−1, −2, and −3). The colors of the letters highlight the physicochemical properties of amino acid residues and are as follows: aliphatic/hydrophobic (pale pink), hydrophilic (green), conformationally special (bright purple), cysteine (yellow), negatively charged (red), positively charged (blue), and aromatic (dull yellow) (Waterhouse et al., 2009). Conserved seven-transmembrane segments (TM1-7) are indicated by black outlined rectangles in the alignment. The consensus sequence is given under the alignment. For GenBank accession numbers, see the caption of Fig. 2.

**Fig. 2:**
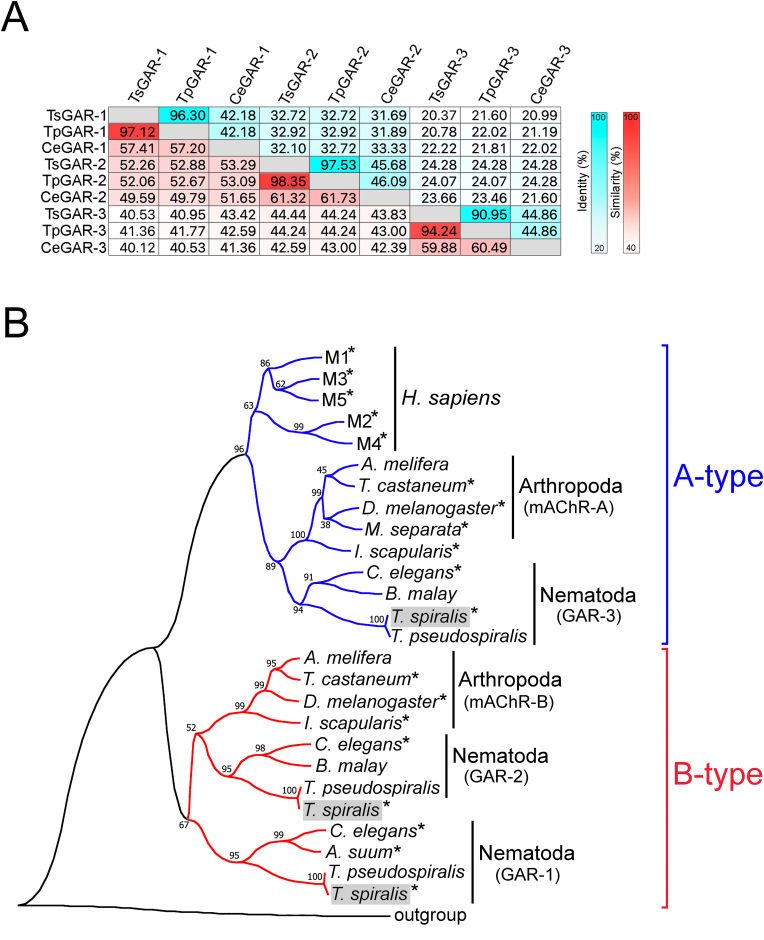
(A) Heat map of protein sequence identities and similarities among the three different GARs from *T. spiralis*, *T. pseudospiralis* and *C. elegans*. Sequences were aligned using DNASTAR software, the MegAlign tool and ClustalW algorithm (Thompson et al., 1994). Aqua blue and red gradients represent the percentage scale of identity and similarity, respectively. (B) Curved neighbor-joining phylogenetic tree derived from human and invertebrate mAChRs. Numbers and nodes are for the percent support in 500 bootstrap replicates. Asterisks indicate deorphanized receptors. The classification of A (blue) and B (red) type mAChRs is based on Ren et al., 2015). The three *T. spiralis* GARs functionally identified in this study are highlighted with a gray background. The sequence for the *Drosophila* short neuropeptide F receptor (sNPF-R) was used as an outgroup. GenBank accession numbers are as follows: *H. sapiens* M1, NP_000729.2; *H. sapiens* M2, NP_001006632.1; *H. sapiens* M3, NP_001362914.1; *H. sapiens* M4, AAM18941.1; *H. sapiens* M5, NP_001307846.1; *D. melanogaster* mAChR-A, AFJ23965.1; *T. castaneum* mAChR-A, AGG09676.1; *A. melifera* mAChR-A, XP_006558421.1, *C. elegans* GAR-3, NP_001024236.1; *M. separata* mAChR-A, KY296116.1; *I. scapularis* mAChR-A, XP_002403135.1; *B. malay* GAR-3, XP_042937257.1; *T. spiralis* GAR-3, KRY27749.1; *T. pseudospiralis* GAR-3, KRY69245.1; *D. melanogaster* mAChR-B, AGE13748.1; *T. castaneum* mAChR-B, AFJ23968.1; mAChR-B *A. melifera*, XP_006558421.1; *I. scapularis* mAChR-B, XP_002416160.3; *C. elegans* GAR-1, NP_001024402.1; *A. suum* GAR-1, ACM78885.1; *T. spiralis* GAR-1, KRY43096.1; *T. pseudospiralis* GAR-1, KRY73645.1; *C. elegans* GAR-2, NP_001022593.1; *B. malay* GAR-2, XP_042932566.1; *T. spiralis* GAR-2, OR220883; *T. pseudospiralis* GAR-2, KRY72904.1; *D. melanogaster* sNPF-R, NP_001262086.1

Homology searches of the *T. spiralis* genome (Korhonen et al., 2016) revealed three putative GARs having an orthologous relationship with those previously identified in *C. elegans* (Hwang et al., 1999; Lee et al., 1999, Lee et al., 2000). The sequences were further analysed to predict gene and protein structures. The full-length ORFs of TsGAR-1, -2, and -3 were found to be intronless and correspond to the computationally predicted proteins GenBank Accession nos. KRY43096, KRY31863, and KRY27749, respectively, obtained from the assembled *T. spiralis* genome (BioProject: PRJNA257433) (Korhonen et al., 2016). Among them, the ORF of TsGAR-3 in our study was also experimentally characterized to confirm sequence identity with KRY27749. The publicly available KRY31863 (TsGAR-2) was found to be wrongly annotated on its N-terminal, containing additional 148 amino acid residues before the first methionine of the protein (Supplementary Fig. S1). The corrected TsGAR-2 has been submitted to GenBank as OR220883. Full-length TsGAR-1, -2, -3 proteins contain the typical GPCR signature of seven transmembrane α-helices (Fig. 1) with 635, 762, and 715 amino acid residues, respectively. We compared the full protein sequences of TsGAR-1, -2, and -3 with those computationally predicted from the *Trichinella pseudospiralis* genome (Korhonen et al., 2016), as well as those experimentally characterized previously in *C. elegans* (Hwang et al., 1999; Lee et al., 1999, Lee et al., 2000). In *C. elegans*, all the GARs are alternatively spliced (Suh et al., 2001; Park et al., 2000, 2003), and for our sequence identity/similarity analysis we used AF117300 for GAR-1, AY053365 for GAR-2 and AF139093 for GAR-3 isoforms. TsGAR-1 showed 96.3% and 42.2% identity with *T. pseudospiralis* GAR-1 and *C. elegans* GAR-1, while the similarity was 97.1% and 57.41%, respectively. TsGAR-2 showed 97.53% identity and 98.4% similarity to *T. pseudospiralis* GAR-2, while identity level to *C. elegans* GAR-2 was 45.7% and similarity 61.3%. The TsGAR-3 protein is 91% and 44.9% identical, and 94.2% and 59.9% similar to *T. pseudospiralis* and *C. elegans* GAR-3, respectively (Fig. 2 A).

Both earlier and more recent studies on nematode GARs have suggested that GAR-1 and GAR-2 are closely related in structure, and are both evolutionarily distinct from human M1-M5 receptors (Lee et al., 1999, Lee et al., 2000; Kimber et al., 2009). On the contrary, GAR-3's sequence was more similar to mammalian mAChRs (Hwang et al., 1999; Gallo et al., 2023). Likewise, a study on *D. melanogaster* and *Tribolium castaneum* mAChR suggested two types of mAChRs (type A and B) in arthropods and most other invertebrate phyla including nematodes, where type A is structurally and pharmacologically similar to mammalian muscarinic receptors, whereas type B seems to be mainly found in invertebrates (Collin et al., 2013; Ren et al., 2015). The same characteristic was recently confirmed for type A and type B mAChRs in *Ixodes scapularis* and *Ixodes ricinus* ticks (Mateos-Hernández et al., 2020).

In the current study, a comparative protein sequence analysis of mammalian, arthropod and nematode mAChRs yielded a phylogenetic tree with two major branches (Fig. 2 B). One branch grouped both nematode GAR-3 and arthropod type A mAChRs, along with mammalian M1-M5. The other branch demonstrated the clear orthologous cluster of nematode GAR-1 and GAR-2, including those of *T. spiralis,* and arthropod type B mAChRs, but without vertebrate/mammalian counterparts. Although the cluster of type A mAChRs (including nematode GAR-3) is close to the M1-M5 clade, there is an obvious evolutionary distance between these two groups. Conversely, the divergence feature of type B mAChRs (including GAR-1 and GAR-2) indicates a common ancestor for the current mAChR family; indeed, an earlier study (Ren et al., 2015) suggested that this is specific to protostomes. The significant evolutionary distance between nematode GAR-1/GAR-2 and mammalian muscarinic receptors reasonably leads to speculation on their different pharmacological profile, which has already been investigated in some nematodes (Lee et al., 1999, Lee et al., 2000; Kimber et al., 2009), thus positioning them as a suitable candidate for selective drug targeting (Gallo et al., 2023).

### 3.2 Stage specific expression of TsGAR-1, -2, and -3

**Fig. 3:**
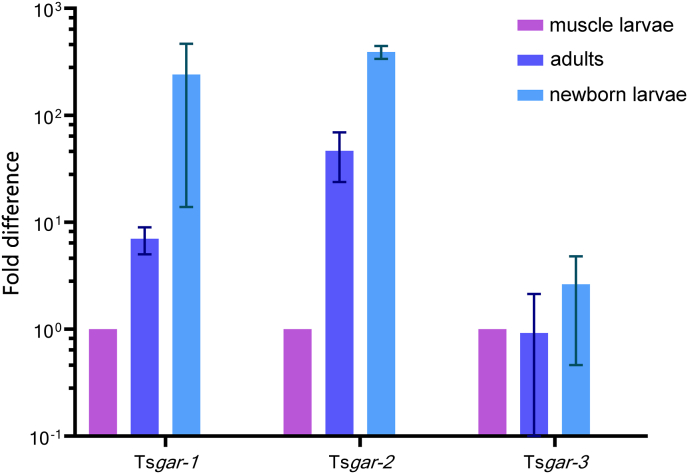
Quantitative RT-PCR showing the transcript levels of *T. spiralis gar-1, -2,* and *-3* in three different life stages. Data were normalized using the eif3C transcript, and the expression levels in muscle larvae were assigned value 1. The averages and standard error bars are shown for two biological replications.

The mRNA of TsGAR-1, -2, and -3 was detected in all investigated stages, including muscle larvae, adults and newborn larvae (Fig. 3). This finding is in agreement with previous studies of *C. elegans* GAR-1, -2 and -3 and *B. malayi* GAR-2 and -3 that were also confirmed to be expressed throughout the entire life cycle (Lee et al., 1999, Lee et al., 2000; Gallo et al., 2023). In case of *B. malayi* the authors observed that GAR-3 mRNA is much highly abundant over the life cycle than in GAR-2, likely reflecting the acute physiological needs of the nematode (Gallo et al., 2023). At this point it would be plausible to investigate if the GARs expression possesses the same feature across different nematode species.

Several other studies confirmed GAR expression in neuronal or non-neuronal tissues. For example, in *C. elegans*, GAR-1 and GAR-2 were found in anterior (head) sensory neurons, though GAR-2 was also identified in a single periventricularis magnocellularis (PVM) neuron (Lee et al., 2000). GAR-1 mRNA in *A. suum* was detected in the head and tail, but not in the dorsal or ventral body wall or ovijector (Kimber et al., 2009). *C. elegans*’ GAR-3 regulates pharyngeal contractions (Steger and Avery, 2004) and is also expressed in protractor muscles, as well as spicule-associated SPC and PCB cholinergic neurons (Liu et al., 2007). GAR-3 in *Brugia malayi* has been found to be expressed in body wall muscle and neurons, as well as digestive and reproductive tissues, largely matching GAR-3 expression in *C. elegans* (Gallo et al., 2023). Thus, it appears that GARs regulate many physiological functions of nematodes, some of which might be conserved across different taxa of this phylum.

### 3.3 *T. spiralis* GAR-1, -2, and -3 couple with G_q/11_ family members when heterologously expressed in CHO cells

More than two decades ago, three GARs were identified in the *C. elegans* genome (Hwang et al., 1999; Lee et al., 1999; 2000). The first one identified, GAR-3, was thought to couple with G_q/11_-mediated downstream signaling when expressed in CHO cells (Hwang et al., 1999), thus activating the phospholipase C that leads to the intracellular IP3/Ca^2++^ cascade, a typical mechanism for mammalian M1, M3, and M5 receptors (Wess, 1996; Caulfield and Birdsall, 1998). The same study also showed that this receptor activates endogenous Cl^−^ currents when expressed in *Xenopus* oocytes, suggesting a coupling with the G_s_ family that mediates the increases in intracellular cAMP levels. Furthermore, the association of *C. elegans'* GAR-3 with calcium signals has also been observed in pharyngeal muscle cells (Steger and Avery, 2004) and neurons controlling protractor muscles (Liu et al., 2007). The functional expression of *C. elegans'* GAR-1 and GAR-2 in the *Xenopus* system has been shown to lead exclusively to the activation of a G protein-activated inwardly rectifying K^+^ (GIRK1) channel (Lee et al., 1999, Lee et al., 2000; Suh et al., 2001). This mechanism mimics the downstream actions of M2 and M4 receptors that are known for coupling with the G_i/0_ family protein and either i) inhibits adenyl cyclase activity via the α_i/0_ subunit, thus decreasing intracellular cAMP levels (McKinney et al., 1991), or ii) opens GIKR1 currents via the βγ complex (Clapham and Neer, 1997). In fruit fly *D. melanogaster,* armyworm *Mythimna separata* and tick *I. scapularis* mAChR-A has been shown to be linked to G_q/11_ supporting the downstream signals of its nematode ortholog GAR-3 (Lü et al., 2020; Mateos-Hernández et al., 2020; Ren et al., 2015). Furthermore, the fruit fly's mAChR-B, which is orthologous to the nematode's GAR-1 and GAR-2, failed to trigger an intracellular IP3/Ca^++^ cascade in CHO cells, and it was suggested that it couples with G_i/0_, based on the cAMP-mediated activities of the cyclic nucleotide-gated channel (CNG) in HEK cells (Ren et al., 2015). However, as in this assay the activity of the receptors were measured exclusively by monitoring calcium influxes through the CNG channels, whether these actions were cAMP- or cGMP–mediated remains to be clarified.

**Fig. 4:**
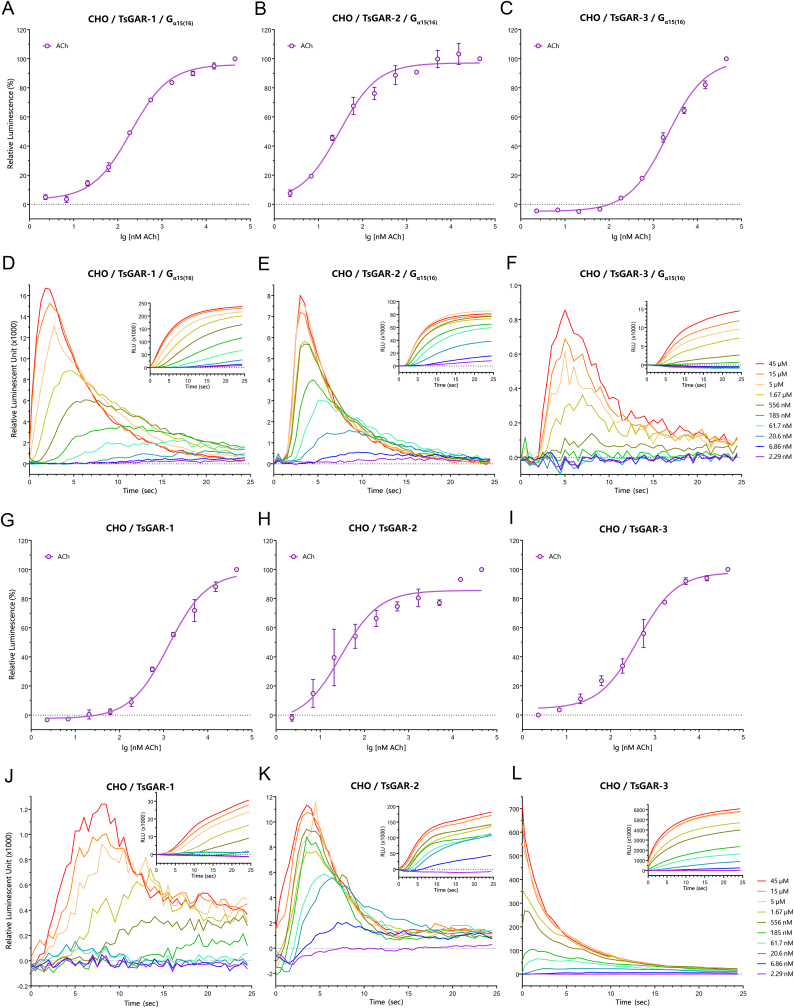
ACh-mediated responses of *T. spiralis'* GAR-1, -2, and -3 measured via intracellular Ca^++^ mobilization in CHO cells with or without G_α15(16)_. Dose-response curves (A–C) and typical 25 s cellular responses (D–F) of ACh-mediated calcium mobilization via TsGARs co-expressed with G_α15(16)_. Dose-response curves (G–I) and typical 25 s cellular responses (J–L) of ACh-mediated calcium mobilization via TsGARs without the presence of G_α15(16)_. The insets in D-F and J-L show integrated values from 2.29 nM to 45 μM ACh concentrations. Note that although all three TsGARs triggered Ca^++^ mobilization regardless of the presence or absence of G_α15(16)_ (A-C and G-I), noticeable qualitative differences between these two conditions were observed in active ACh doses during the 25 s intervals (D-F and J-L). The bars indicate the standard error for two biological replications. If standard error bars are smaller than the symbols used, in these cases, only the symbols are shown.

In the present study, we functionally identified three TsGARs in an assay using CHO cells with aequorin by monitoring changes in intracellular calcium mobilization upon activation of the receptor (Fig. 4). First, to ensure that our transfected plasmid constructs form functional receptor proteins in our heterologous expression system, we co-expressed each of the TsGARs with promiscuous G_α15(16)_, which is known to link G_i/0_ and G_s_ coupled GPCRs to the intracellular calcium mobilization pathway (Offermanns and Simon, 1995; Park et al., 2002). In this experimental setup, all three TsGARs showed dose-dependent activity when exposed to different concentrations of ACh ligand (Fig. 4 A-F). Interestingly, when the TsGAR-1, -2, and -3 were expressed alone (without G_α15(16)_), ACh also mediated effective responses via all three TsGARs (Fig. 4 G-L). Here our results regarding the TsGAR-3 G_q/11_–mediated signaling pathway are in agreement with those suggested for *C. elegans'* GAR-3 as well as its ortholog mAChR-A in *Drosophila* (Hwang et al., 1999; Ren et al., 2015). However, the ability of TsGAR-1 and TsGAR-2 to trigger the G_q/11_ pathway, as found in this study, does not entirely support the proposed mode of action of *C. elegans*’ GAR-1 and GAR-2 along their *Drosophila* mAChR-B ortholog that were thought to couple with the G_i/0_ family (Lee et al., 1999, Lee et al., 2000; Ren et al., 2015). One possible explanation of these discrepancies is that downstream signals depend on different types of cell, and potential cross-talk between signaling pathways, as these features are gradually becoming accepted as being of importance among the mAChR signal transduction mechanisms (Nathanson, 2000). To confirm this assertion, it would be plausible to test whether GAR-1 and GAR-2 from *T. spiralis* and *C. elegans* act in the same way either in CHO or *Xenopus* expression systems. However, it is important to highlight that although transfection of either CHO, HEK or an insect Sf9 cell line with *A. suum* GAR-1 produced the representative mRNAs, no functional protein was formed (Kimber et al., 2009). We wonder if in our study the TsGAR-1 and TsGAR-2 codon optimisation for a mammalian system enhanced the production of these proteins, leading to a successful assay testing these receptors in our experiments.

Although our data clearly indicate that all three *T. spiralis* GARs couple with the G_q/11_ family members in CHO cells, significant qualitative differences were observed in the 25 s responses of active doses of ACh ligand depending on the presence/absence of G_α15(16)_ (Fig. 4 D-F, J-L). Specifically, in the case of TsGAR-1 and TsGAR-2, the presence of G_α15(16)_ resulted in more coherent ACh dose-responses (Fig. 4D and E) than the absence of G_α15(16)_ (Fig. 4 J, K). In addition, the increase in luminescence elicited by TsGAR-1 activities was slower when G_α15(16)_ was absent (Fig. 4 J), while nearly immediate responses were detected when present (Fig. 4 D). The features thus observed may indicate a complex interaction between either TsGAR-1 or TsGAR-2 on the one hand and endogenous signaling G protein(s) in CHO cells on the other. Interestingly, TsGAR-3 was more sensitive to different ACh doses and was immediately responsive when expressed without G_α15(16)_ (Fig. 4 F, L), suggesting its endogenous coupling with G_q/11_.

In either case, our study revealed that the hypothesis put forward by previous authors (Ren et al., 2015), whereby all the protostome B-type mAChRs are linked with the G_i/0_ pathway does not appear to be absolute and more studies are required to clarify the downstream coupling of this family of receptors.

### 3.4 Pharmacology of TsGAR-1, -2, and -3

**Fig. 5:**
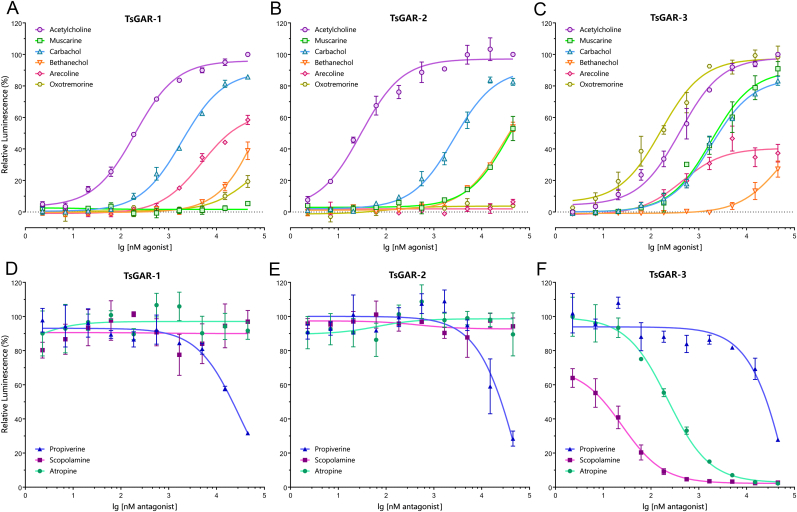
Dose-response curves of the muscarinic agonistic (A–C) and antagonistic (D–F) effects on *T. spiralis'* GAR-1, -2, and -3. The bars indicate the standard error for a minimum of three biological replications. If standard error bars are smaller than the symbols used, in these cases, only the symbols are shown. For the EC_50_ and IC_50_ values of tested drugs see Table 1. Note that TsGAR-1 and TsGAR-2 were co-expressed with G_α15(16)_, but TsGAR-3 was not.

The pharmacology of invertebrate mAChRs, namely GARs in nematodes, has been investigated by different research groups in various expression systems (Hwang et al., 1999; Lee et al., 1999, Lee et al., 2000; 2000; Suh et al., 2001; Kimber et al., 2009; Collin et al., 2013; Ren et al., 2015; Mateos-Hernández et al., 2020; Gallo et al., 2023). The take-home message of this research is that invertebrates possess two types of mAChRs: the single type A (an ortholog of GAR-3) is pharmacologically close to the mammalian type of receptor, while type B (orthologs of GAR-1 and GAR-2) had an atypical muscarinic profile (Collin et al., 2013; Ren et al., 2015). Our study tested six muscarinic agonist and three antagonists for their affinity to *T. spiralis* GAR-1, -2, and -3 (Fig. 5 A-F and Table 1). To achieve the highest sensitivity of each receptor to the ligands, both TsGAR-1 and TsGAR-2 were tested in the presence of G_α15(16)_, while TsGAR-3 was tested without (see section 3.3 in Results and Discussion). In this assay setting, ACh was a potent activator of all three receptors, with EC_50_ values of 194.5, 14.67 and 400.3 nM respectively. This assay was followed by a non-selective cholinergic agonist, carbachol, which gave an EC_50_ of 2047, 2668 and 1935 nM respectively (Fig. 5 A-C and Table 1). Interestingly, the activities of the classical muscarinic agonist oxotremorine were barely noticeable on TsGAR-1, and virtually no response was detected for TsGAR-2 (Fig. 5 A, B and Table 1). These results are similar to those produced in *C. elegans*, where this drug activated both GAR-1 and GAR-2 with very little effect (Lee et al., 1999, Lee et al., 2000). However, oxotremorine was effective in activating GAR-1 in parasitic nematode *A. suum* (Kimber et al., 2009). In the case of TsGAR-3, oxotremorine showed about 2.8 times higher potency than ACh, with an EC_50_ value of 138.8 nM (Fig. 5 C and Table 1). Completely novel results were produced when TsGAR-1, -2, and -3 were tested for their affinity to muscarine, an agonist recognized as the primary distinguisher between mAChR groups and nAChR groups (Milsom, 2007). Surprisingly, TsGAR-1 and TsGAR-2 showed no or low affinity to this drug, respectively (Fig. 5 A, B, and Table 1), while muscarine was a potent agonist for TsGAR-3 with response levels close to those of carbachol, reaching an EC_50_ value of 2495 nM (Fig. 5 C and Table 1). Intriguingly, in both *D. melanogaster* and *T. castaneum*, muscarine showed affinity to mAChR-B, respectively, with about 1000 and 100 times lower sensitivity than to mAChR-A (Collin et al., 2013). Thus, it appears that nematode receptors GAR-1 and GAR-2 are less sensitive to muscarine than their arthropod mAChR-B orthologs. The agonist arecoline was found to effectively activate *A. suum* GAR-1 (Kimber et al., 2009), and in our study was identified as a partial agonist of TsGAR-1 and TsGAR-3 with an EC_50_ of 4632 and 457.4 nM, respectively (Fig. 5 A, C and Table 1). Subsequently, we did not detect any arecoline-triggered activation of TsGAR-2 (Fig. 5 B and Table 1). Bethanechol's agonistic effect was shown in the case of *A. suum* GAR-1 (Kimber et al., 2009), but in our assays low potency was observed when TsGAR-1, -2, and -3 were exposed to high concentrations of this drug (Fig. 5A–C and Table 1).

**Table 1:** EC_50_ and IC_50_ (nM) for different agonistic and antagonistic ligands, respectively, on three different *T. spiralis* GAR-1, -2, and - 3. n_H_ – Hill coefficient. ND – not detectable. Note that standard errors (SE) are shown for three biological replications.

	TsGAR-1		TsGAR-2		TsGAR-3	
Agonist	EC_50_ ± SE (nM)	n_H_	EC_50_ ± SE (nM)	n_H_	EC_50_ ± SE (nM)	n_H_

Acetylcholine	194.5 ± 18.10	0.8621	14.67 ± 10.77	0.5883	400.3 ± 73.14	0.7319
Muscarine	ND	–	> 10^4^	0.8666	2495 ± 960.3	0.7106
Carbachol	2047 ± 235.2	0.8693	2668 ± 507.9	0.9531	1935 ± 255.8	0.9117
Bethanechol	> 10^4^	0.9289	> 10^4^	0.8738	> 10^4^	1.390
Arecoline	4632 ± 840.8	1.054	ND	–	457.4 ± 156.1	1.199
Oxotremorine	> 10^5^	1.712	ND	–	138.8 ± 39.99	0.6856
**Antagonist**	**IC_50_ ± SE (nM)**	**n**_**H**_	**IC_50_ ± SE (nM)**	**n**_**H**_	**IC_50_ ± SE (nM)**	**n**_**H**_
Propiverine	> 10^4^	−0,6695	> 10^4^	−3.002	> 10^4^	−0.6649
Scopolamine	ND	–	ND	–	27.3 ± 6.296	−1.151
Atropine	ND	–	ND	–	222.3 ± 42.75	−0.8748

In our antagonistic assays, the activities of TsGAR-1, -2, and -3 were partially blocked by high concentrations of propiverine (Fig. 5 D-F and Table 1). This finding contrasts slightly with those for *C. elegans* GAR-1 and GAR-2 as well as *A. suum* GAR-1, given that this drug was observed to have largely no effect on these receptors’ activities (Lee et al., 1999, Lee et al., 2000; Kimber et al., 2009). The classical muscarinic antagonists scopolamine and atropine were not effective in blocking TsGAR-1 and TsGAR-2 activities (Fig. 5 D, E and Table 1), while both drugs effectively inhibited TsGAR-3 with an IC_50_ of 27.3 and 222.3 nM, respectively (Fig. 5 F and Table 1). In the same expression system as in our study, the activities of *D. melanogaster* and *T. castaneum* mAChR-Bs also remained unaffected by scolopamine and atropine (Collin et al., 2013). On the other hand, in both *C. elegans* and *A. suum*, atropine was effective in inhibiting GAR-1 while scopolamine was not (Lee et al., 1999; Kimber et al., 2009).

Our investigation confirms that both TsGAR-1 and TsGAR-2 have an atypical muscarinic pharmacological profile that is different from that of TsGAR-3, a receptor which appears to be pharmacologically closer to mammalian and invertebrate type A muscarinic receptors (Kimber et al., 2009; Lee et al., 2000; 1999; Ren et al., 2015). As discussed in an earlier study (Kimber et al., 2009), this difference might be caused by altered key amino acid variations in transmembrane domains or intra/extracellular loops critical for ligand-receptor binding and/or structural receptor conformation and cell signaling (Burstein et al., 1996; Heitz et al., 1999, 1999, 1999; Hulme et al., 2001; Maeda et al., 2020). Furthermore, we clearly demonstrated pharmacological differences between TsGAR-1 or TsGAR-2 and their orthologs in *C. elegans* and *A. suum*. The most noticeable difference appears to be the action of conventional muscarinic antagonist atropine, which effectively blocked both *C. elegans* and *A. suum* GAR-1 (Lee et al., 1999; Kimber et al., 2009), but was totally ineffective in inhibiting TsGAR-1. Another example is oxometrine's high potency in *A. suum* GAR-1 activation (Kimber et al., 2009) and its barely noticeable effect in *C. elegans* as well as TsGAR-1 tested in our study. At this point, it is difficult to speculate whether these discrepancies are receptor-specific or caused by a variety of receptor expression systems used by different research groups. In either case, our results shed further light on nematode GAR pharmacology and suggest that GAR-1 and GAR-2 could be suitable candidates for selective drug targeting.

## Declarations of interest

None.
