# Supplementary material for: Functional characterization of three G protein-coupled acetylcholine receptors in parasitic nematode Trichinella spiralis
Source: Int J Parasitol Drugs Drug Resist. 2023 Nov 24;23:130–9. doi: 10.1016/j.ijpddr.2023.11.005 (PMC10731000; doi:10.1016/j.ijpddr.2023.11.005)
Supplement: Multimedia component 1 [file mmc1.docx]

**Functional Characterization of Three G Protein-Coupled Acetylcholine Receptors in Parasitic Nematode *Trichinella spiralis***

Cáinà Nìng^1^, Aurélie Heckmann^1^, Lourdes Mateos-Hernandez^1^, Grégory Karadjian^1,2,*^, Ladislav Šimo^1,2,*^

^1^Laboratoire de Santé Animale, UMR BIPAR, Ecole Nationale Vétérinaire d’Alfort, INRAE, ANSES, F-94700 Maisons-Alfort, France

^2^Equal contribution

^*^Corresponding author: [gregory.karadjian@anses.fr](mailto:gregory.karadjian@anses.fr), [ladislav.simo@vet-alfort.fr](mailto:ladislav.simo@vet-alfort.fr)

Key words: Trichinella, GAR receptors, CHO cells, pharmacology

KRY31863.1 MWKAMSMLILNEPINNANFITHINFLIIFTRIPTVSFYASFYFPFVDMKSLKLFHCFHIT 60

OR220883 ------------------------------------------------------------ 0

KRY31863.1 FKRQYSKSIRVKKYQGHLTFVLKLNYTFSKTWLVVHARAVFFNDHPSHLSTFFRKMLPFL 120

OR220883 ------------------------------------------------------------ 0

KRY31863.1 SILSSQWAIHIGNLPIKESLLISKLVFKMNPAIGTIDMPALNVNHTLRNSTDANFDWSSP 180

OR220883 ----------------------------MNPAIGTIDMPALNVNHTLRNSTDANFDWSSP 32

********************************

KRY31863.1 YSLTEIIILGMITASLSIITVVGNLMVMISFYIDKNIRQPSNYFIFSLAVSDFLIGLEGF 240

OR220883 YSLTEIIILGMITASLSIITVVGNLMVMISFYIDKNIRQPSNYFIFSLAVSDFLIGLEGF 92

************************************************************

KRY31863.1 PLYSIYVLNGQKWTMGWFLCDLWLSVDYSACLASTYTVLFITIDRYCSVKIPTTYRNWRT 300

OR220883 PLYSIYVLNGQKWTMGWFLCDLWLSVDYSACLASTYTVLFITIDRYCSVKIPTTYRNWRT 152

************************************************************

KRY31863.1 QRKVLVIIAITWLVPTLLFFISVFGWGYFSGQGRVLAEHECMVQFMVDPYFNMSMYISYY 360

OR220883 QRKVLVIIAITWLVPTLLFFISVFGWGYFSGQGRVLAEHECMVQFMVDPYFNMSMYISYY 212

************************************************************

KRY31863.1 WSTLIVMIILYAGIYRAARNLHLKSKQKRQRFQAICALRATTTPLTMKSSTLKEEDDSSQ 420

OR220883 WSTLIVMIILYAGIYRAARNLHLKSKQKRQRFQAICALRATTTPLTMKSSTLKEEDDSSQ 272

************************************************************

KRY31863.1 ITPEHSEGSSGAQHAKTPAGTSAVAKSSRNHTSAKQSSSAAAKGKALLPAVNNISSSSAC 480

OR220883 ITPEHSEGSSGAQHAKTPAGTSAVAKSSRNHTSAKQSSSAAAKGKALLPAVNNISSSSAC 332

************************************************************

KRY31863.1 STDESDAAKMQNKVQPSDSSQSQYDNMPKEKKEEALCENLDNLIPLEKSVSFMNETNNSC 540

OR220883 STDESDAAKMQNKVQPSDSSQSQYDNMPKEKKEEALCENLDNLIPLEKSVSFMNETNNSC 392

************************************************************

KRY31863.1 NSPPNGDVPNPKSTAMSVILKRNRLSEPECHVVLAEEAMKATKRNLSLNFNRIADIDSDY 600

OR220883 NSPPNGDVPNPKSTAMSVILKRNRLSEPECHVVLAEEAMKATKRNLSLNFNRIADIDSDY 452

************************************************************

KRY31863.1 PSLKSPEEFFFLDIALKETVSPKSLSPSASGSVRMDQEPTLLYANAASTLPYVDANVKPQ 660

OR220883 PSLKSPEEFFFLDIALKETVSPKSLSPSASGSVRMDQEPTLLYANAASTLPYVDANVKPQ 512

************************************************************

KRY31863.1 SDNDHNSTEPNCQVASLAKNECAWETILEISPERMSKIAQPKSNDIKQESDEEDFSSHRN 720

OR220883 SDNDHNSTEPNCQVASLAKNECAWETILEISPERMSKIAQPKSNDIKQESDEEDFSSHRN 572

************************************************************

KRY31863.1 IIKNAGIIYEHEGHERRWTFGRFQIRWEYHPGFFQKSPKVKKPVESKEDTNDEAKKSDTM 780

OR220883 IIKNAGIIYEHEGHERRWTFGRFQIRWEYHPGFFQKSPKVKKPVESKEDTNDEAKKSDTM 632

************************************************************

KRY31863.1 TADTAKLLPHAGLVQASKATVSRLFGRMPTRQAGSSNSLMYRKSKSENRARKALRTITVI 840

OR220883 TADTAKLLPHAGLVQASKATVSRLFGRMPTRQAGSSNSLMYRKSKSENRARKALRTITVI 692

************************************************************

KRY31863.1 LGAFVAFWTPFYVLATIYGFCEDCVPKTVYVVSYYLCYMNSPINPFCYALANVQFKKTLS 900

OR220883 LGAFVAFWTPFYVLATIYGFCEDCVPKTVYVVSYYLCYMNSPINPFCYALANVQFKKTLS 752

************************************************************

KRY31863.1 RMLKGDFHRT 910

OR220883 RMLKGDFHRT 762

**********

Supplementary Figure S1. Protein sequence alignment of TsGAR-2. KRY31863.1 represents the incorrectly predicted protein on the N-terminal (red), while OR220883 corresponds to the corrected sequence used in this study.
